# A Retrospect of the Special Issue “Second Edition of Innovative Solutions for Oral Healthcare”

**DOI:** 10.3390/healthcare11131952

**Published:** 2023-07-06

**Authors:** Saturnino Marco Lupi

**Affiliations:** Department of Clinical Surgical, Diagnostic and Pediatric Sciences, School of Dentistry, University of Pavia, 27100 Pavia, Italy; saturninomarco.lupi@unipv.it

Medicine and oral health are constantly progressing through a series of small advancements that, together, lead to significant discoveries and breakthroughs. These achievements enable the management of an increasing number of diseases and improve the quality of care and rehabilitation in the field of dentistry. In the present text, the content of this Special Issue, titled “Second Edition of Innovative Solutions for Oral Healthcare”, is summarized, which comprises a variety of studies, clinical cases, and reviews aimed at providing new knowledge in the field of oral health and its related topics. The articles cover subjects such as early diagnosis, technological innovation, student education, the use of new therapies, and the impact of the environment on oral health. The objective is to continue promoting research and innovation in the field of dentistry.

In the first volume of this Special Issue, oral health was linked to physiological alterations, aging, early diagnosis, environmental pollution, student education, digital dentistry, and the application of new technologies. Ikebuchi et al. [1] identified a relationship between masticatory function and bone mineral density. Mohamed et al. [2] identified a valuable and cost-effective tool that utilizes artificial neural networks based on patterns of volatile organic compounds present in the breath to enable the early diagnosis of oral squamous cell carcinoma in limited-resource settings.

An important aspect of the published research was evaluating the quality of training for students and recent graduates, both in terms of their ability to diagnose benign tumors, malignant tumors, and premalignant lesions through a cross-sectional study [3], and in terms of their psychological distress levels through a systematic review [4], which identified higher levels of depression, stress, and anxiety during their education period. Additionally, one study focused on the student’s ability to adequately perform complex therapies such as orthodontics [5].

New drugs are constantly being proposed, influencing the management of patients undergoing oral surgery. In the case of patients taking direct oral anticoagulants, a peri-operative management protocol has been proposed based on an analysis of the existing literature [6].

In the current Special Issue, many other important topics have been addressed. Moga et al. [7] explored the theme of root resorption as a side effect of orthodontic treatment using Finite Element Analysis (FEA) based on Tresca criteria. Dragus et al. [8] assessed sagittal condylar inclination using an ultrasonic jaw tracking device, providing valuable information for both gnathologists and prosthodontists. Alwadei et al. [9] identified risk factors for sleep-disordered breathing in 1866 children aged 6–12 years. Alamri et al. [10] tested a new minimally invasive method to detect vertical root fractures.

Implant-prosthetic rehabilitation is at a turning point compared to in the past: from traditional techniques based on the manual fabrication of prostheses, it is now possible to accurately predict and plan the rehabilitation, which will then be executed with the highest precision and predictability. This is made possible by the ever-increasing diagnostic possibilities (CBCT, facial scan, and optical impressions), extremely advanced CAD/CAM technologies, and innovative materials (zirconia, PEEK, and PMMA). In this Special Issue, two studies address this topic.

Todaro et al. [11] presented a comprehensive digital implant-supported complex rehabilitation process that included bone regeneration, guided implant positioning, facial scans, digital smile design, and leukocyte–platelet-rich fibrin. In this study, the most current technologies were applied to achieve implant-prosthetic rehabilitation in a case of severe bone resorption, demonstrating how therapeutic possibilities are being expanded through the application of new techniques.

Azpiazu-Flores et al. [12] presented a case of complex implant-prosthetic rehabilitation using innovative CAD/CAM techniques that allowed for highly accurate planning and diagnosis. The final rehabilitation was achieved with a prosthesis composed of a titanium bar and a zirconia overlay.

Based on the considerations mentioned above, the Guest Editor of the present Special Issue would like to thank all clinicians and researchers who contributed their relevant manuscripts.
